# Data-Driven Thyroid Nodule Diagnosis Using Belief Rule Base

**DOI:** 10.3390/diagnostics12102299

**Published:** 2022-09-23

**Authors:** Jiang Jiang, Ruirui Zhao, Xuan Li, Leilei Chang

**Affiliations:** 1 College of Systems Engineering, National University of Defense Technology, Changsha 410073, China; 2 School of Automation, Hangzhou Dianzi University, Hangzhou 310018, China; 3 Department of Ultrasound, The First Affiliated Hospital of USTC, Hefei 230002, China

**Keywords:** data-driven, thyroid nodule diagnosis, belief rule base

## Abstract

Doctors’ diagnosis preferences are different, which makes them adopt different assumptions in medical decision making. Taking the diagnosis of thyroid nodules as an example, this study compares three assumptions, namely deletion, imputation based on the distribution (distribution), and benign by default (benign). For deletion, which is the most used assumption, the clinical reports with missing features would be deleted. For distribution, the missing features would be replaced with a distribution of features with respective probabilities. Besides the two assumptions, certain doctors have also stated that they leave benign features unrecorded because they think that such benign features are irrelevant to the final diagnosis. Under the benign assumption, the missing features would be replaced with benign features. The three assumptions are tested comparatively. Moreover, the belief rule base (BRB) is used to construct the diagnostic model under the three assumptions since it is essentially a white-box approach that can provide good interpretability and direct access to doctors and patients. A total of 3766 clinical reports on thyroid nodule diagnosis were collected from ten radiologists over a seven-year period. Case study results validate that the benign by default assumption has produced the optimal results, although different doctors could present varied tendencies towards different assumptions. Guidance and suggestions for doctors’ practical work have been made based on the study results to improve work efficiency and diagnostic accuracy.

## 1. Introduction

Data are the interface between the practical real-world systems and the constructed model [1]. As uncertainty generally exists in practices, it is important to conduct accurate medical diagnoses using uncertain data [2]. Take the diagnosis of thyroid nodules as an example. There is always missing information in the clinical reports of thyroid nodules by doctors, which causes uncertainty in medical diagnosis [3]. In this sense, it is fair to assert that most, if not all, medical diagnosis is based on incomplete information, but not complete information.

Thyroid nodules are a common medical condition; however, they could be directly related to thyroid cancer if not accurately diagnosed in time and well-treated. The incidence of thyroid nodules increases with age, which is a serious threat to human health [4]. The American Thyroid Association (ATA) defines a thyroid nodule as “a thyroid scattered lesion that can be clearly distinguished from the surrounding thyroid tissue” [5]. Imaging examination plays an irreplaceable role in thyroid nodule diagnoses, such as computed tomography (CT), magnetic resonance imaging (MRI), and ultrasound [6]. Among them, CT and MRI, although far more expensive, cannot well distinguish whether there is airway compression or the extent of the posterior sternal thyroid gland [7]. Comparatively, the much cheaper ultrasound is more suitable for thyroid nodule diagnosis [8,9].

There are two major concepts that need to be cleared, namely the criterion and feature. A **criterion** in thyroid nodule diagnosis is one aspect that needs to be carefully observed. Normally, there are five major criteria, namely margin, contour, echogenicity, calcification type, and vascular distribution [10]. A **feature** in thyroid nodule diagnosis is the specific condition of a patient concerning one criterion [11]. For example, a feature presented in a patient concerning the “margin” criterion could be “well-defined (towards benign)” or “ill-defined (towards malignant)”. Nevertheless, a cutting-edge technique can only help provide high-resolution images, but doctors still need to translate those images into features concerning different criteria and record them in clinical reports [7,12]. However, it has to be found that missing features generally exist in many clinical reports. There could be different reasons for the missing features, e.g., lack of proper testing equipment, improper data-gathering/transference/handling practices, or simply the inability of the doctors [13,14]. Nevertheless, such missing features have brought great uncertainty in thyroid nodule diagnosis and should be properly addressed [15,16].

Although doctors can well understand the clinical reports with missing features as they are, they can not be handled in a diagnostic model or approach because they would be in incomplete format. As a consequence, the clinical reports with missing features would be deleted, which is common practice. However, after deletion, the remainder may be insufficient to generate statistically significant results [17,18]. To properly handle such missing features, researchers from different backgrounds have proposed different approaches. Among them, imputation is another type of approach that has been widely used [19]. In short, the imputation approach would fill the missing features with imputed features using the information in all clinical reports [20,21]. The original clinical reports must be of a large size to support the statistical calculation required by imputation [22,23,24].

To summarize, both the deletion and imputation approaches require a large amount of data [25,26,27]. However, it may be unsuitable to certain practical conditions when a large amount of data are unavailable. Moreover, the characteristics of the thyroid nodule diagnosis problem should also be taken into consideration. Certain features may be unrecorded for plausible reasons: they are deemed benign and irrelevant to the final diagnostic result and thus are not recorded. If so, deleting such clinical reports would be a waste of resources and imputation would cause more errors since the missing features should be benign [13,14].

Thyroid nodule diagnosis also requires higher interpretability and transparency, which cannot be provided by many black-box approaches [28]. To meet this challenge, the belief rule base (BRB) expert system is adopted, since it is essentially a white box that can be directly accessed by doctors, patients, and other stakeholders [29,30]. With BRB as the diagnostic tool, the above three assumptions and corresponding handling techniques are explored in this study, including deletion, imputation based on the distribution (“distribution” for short), and benign by default (“benign” for short). For the deletion assumption, the clinical reports with missing features would be deleted. For the distribution assumption, the missing features would be replaced by a distribution of features with respective probabilities. For the benign assumption, the missing features would be replaced by benign features.

To summarize, the primary objective of this paper is to propose a diagnostic approach for thyroid nodules using the belief rule base. Furthermore, three assumptions are tested in handling missing information in thyroid nodule diagnosis, namely deletion, imputation, and benign by default. A case of thyroid nodule diagnosis is studied to validate the proposed approach. A total of 3766 cases were collected from ten doctors in the Department of Ultrasound, The First Affiliated Hospital of USTC, from January 2012 to February 2019. Among them, 3082 cases were used as training data and 684 cases were used as testing data. Case study results show that for most doctors, the benign by default assumption produced the smallest error. Further exploration has also revealed that different doctors have presented varied tendencies, which can be used to identify specific models for different doctors and propose customized work guidance.

The remainder of this study is organized as follows. Section 2 presents the problem formulation and reviews the present literature. Section 3 introduces preliminaries on BRB and its inferencing and optimization procedures. Section 4 further introduces the diagnosis approach under the three assumptions. Section 5 introduces a practical thyroid nodule diagnosis case. This study is concluded in Section 6.

## 2. Literature Review

This section presents a literature review on how to handle the uncertainty that is caused by missing features, especially in the context of thyroid nodule diagnosis.

From the perspective of building a diagnostic model or proposing a diagnostic approach, the most commonly used means to handle missing features in thyroid nodule diagnosis is just to delete the clinical reports with missing features. It is the most effortless method and could be easily implemented, which is an essential factor in daily practice [21]. However, applying the deletion approach requires that the original clinical reports must be in a large size or there would not be enough left after deletion, thereby not meeting the statistical requirement of building a diagnostic model or proposing a diagnostic approach [22,25].

Another commonly used approach is to impute the missing features from the original clinical reports, which is a wider topic and involves more techniques [25]. There are several imputation methods, including mean imputation, regression imputation, hot and cold deck imputation, multiple imputations, etc. Mean imputation replaces missing features with the mean value of the known features [27]. Mean imputation usually underestimates the variance of the imputed missing features [21]. Therefore, it is suitable for simple descriptive research other than for complex analysis of variance estimates. Regression imputation is the regression of missing features to the observations, replacing the missing features with the predicted features [25,26]. The estimated missing features are completely related to the remaining features in the model, which results in multicollinearity [22,24]. This method makes better use of the information provided by the cases. The disadvantage is that all estimates follow a regression curve and do not represent any inherent changes in the cases. Hot deck imputation replaces missing features with similar full features in the current case, and cold deck imputation uses different cases, e.g., historical cases, etc. [20,23]. This method can help avoid the underestimation of variance. The disadvantage is that estimating missing features based on a single complete case lacks consideration of the overall properties of the cases. Multiple imputation (MI) is performed by constructing *n* (*n* > 1) substitute features for each missing feature to form D complete cases [27]. Each complete case is analyzed using the same statistical method for the complete case, and the individual results are combined to produce the final statistical inference. Compared with the single imputation, MI can better reflect the uncertainty caused by missing features and increase the efficiency of estimation.

To summarize, for either deletion of the clinical reports with missing features or imputation from existing clinical reports using whatever techniques, it is required that the size of the original clinical reports should be large enough to meet the requirements. Without a large number of clinical reports, there would be no statistical significance, and the deletion and imputation approaches could not be applied [21,22].

## 3. Preliminaries

### 3.1. BRB Basics

Interpretability is another requirement in constructing a diagnostic model for thyroid nodules. To meet this challenge, the belief rule base (BRB) is applied. As a rule-based expert system, its basic form can be easily understood by people from different professional backgrounds [29]. Its inferencing and integration process is analytic and transparent so that it can be observed, explained, and accessed by experts. Overall, BRB is essentially a white-box approach that can provide direct access to doctors, patients, and other stakeholders [30]. The above characteristics, especially being a white-box approach, makes it particularly suitable for a problem with such requirements, e.g., in the medical diagnosis context with a large economic, strategic, and even life–death significance where decisions must be made after calculated deliberations and the complete decision-making process must be clear to doctors and patients so that the accountabilities can be held [13,14]. It has also been successfully applied in many medical-related diagnoses and assessment problems [31,32,33,34,35].

A belief rule [29] in BRB is given in (1):

(1) $$\begin{array}{l} R_{l}:\mathrm{if}\;(x_{1}\;\mathrm{is}\;A_{1}^{k})\wedge (x_{2}\;\mathrm{is}\;A_{2}^{k})\wedge \cdots \wedge (x_{M}\;\mathrm{is}\;A_{M}^{k})\begin{array}{c} , \end{array}\\ \;\;\;\;\;\;\mathrm{then}\{(D_{1},\beta_{1,l}),\cdots ,(D_{N},\beta_{N,l})\}\;\mathrm{with}\;\mathrm{rule}\;\mathrm{weight}\;\theta_{l} \end{array}$$

where $x_{m}(m=1,\ldots ,M)$ stands for the *m*th attribute, $A_{m}^{k}(m=1,\cdots ,M;k=1,\cdots ,K)$ for the *k*th reference value of the *m*th attribute, *M* for the number of attributes, $\beta_{n,l}(n=1,\ldots ,N)$ for the belief degree for the *n*th assessment grade $D_{n}$, *N* for the number of diagnostic category, and “∧” for the conjunctive operator, which could also be disjunctive “∨” depending on the correlation among the attribute.

### 3.2. BRB Inferencing and Integration Procedures

The first step of BRB inferencing is to calculate the matching degree of the input information with the reference value [29,30]. The matching degree of the input with the *k*th reference value of the *m*th attribute is calculated in Equation (2):

(2) $$\varphi (I_{m}^{*},A_{m}^{j})=\{\begin{array}{ll} \frac{A_{m}^{k+1}-I_{m}^{*}}{A_{m}^{k+1}-A_{m}^{k}} & j=k(A_{m}^{k}\leq I_{m}^{*}\leq A_{m}^{k+1}),\kappa (I_{m}^{*},A_{m}^{j})=1\\ \frac{I_{m}^{*}-A_{m}^{k}}{A_{m}^{k+1}-A_{m}^{k}} & j=k+1,\kappa (I_{m}^{*},A_{m}^{j})=1\\ \;0 & j=1,2,\ldots ,p(m),j\neq k,k+1,\kappa (I_{m}^{*},A_{m}^{j})=0 \end{array}$$

The integrated matching degree for the *m*th attribute in the *l*th rule is calculated in Equation (3):

(3) $$\alpha_{m,l}=\frac{\varphi (I_{m}^{*},A_{m}^{j})\varepsilon_{m}}{\sum \varphi (I_{m}^{*},A_{m}^{j})}$$

where $\varepsilon_{m}$ denotes the belief of the *m*th attribute being assessed as $I_{x}^{*}$.

The integrated matching degree for the *l*th conjunctive rule, $\alpha_{l}$, is calculated by Equation (4):

(4) $$a_{l}=\prod_{m=1}^{M}\alpha_{m,l}$$

The integrated matching degree for the *l*th disjunctive rule [36,37], $\alpha_{l}$, is calculated by Equation (5):

(5) $$a_{l}=\sum_{m=1}^{M}\alpha_{m,l}$$

The activated weight for the *l*th rule, $w_{l}$, is calculated by Equation (6):

(6) $$w_{l}=\frac{\theta_{l}}{\sum_{l=1}^{L}\theta_{l}\alpha_{l}}$$

After BRB inferencing, with $w_{l}$ for *L* activated rules and $\beta_{n,l}$ in *N* diagnosis categories of *L* activated rules, *L* activated rules could be integrated using the Evidential Reasoning (ER) algorithm [38].

(7) $$\beta_{n}=\frac{\mu [\prod_{l=1}^{L}\left(w_{l}\beta_{n,l}+1-w_{l}\sum_{n=1}^{N}\beta_{n,l}\right)-\prod_{l=1}^{L}\left(1-w_{l}\sum_{n=1}^{N}\beta_{n,l}\right)]}{1-\mu [\prod_{l=1}^{L}\left(1-w_{l}\right)]}$$

and

(8) $$\mu ={\left[\sum_{n=1}^{N}\prod_{L=1}^{L}\left(w_{L}\beta_{n,l}+1-w_{l}\sum_{n=1}^{N}\beta_{n,l}\right)-(N-1)\prod_{l=1}^{L}\left(1-w_{l}\sum_{n=1}^{N}\beta_{n,l}\right)\right]}^{-1}$$

where $\beta_{n}$ represents the integrated belief for the *n*th diagnosis category.

### 3.3. BRB Optimization Procedures

As the initial BRB is constructed based on experts’ knowledge and historical data, it could be quite inaccurate, which calls for constructing an optimization model and designing an optimization algorithm.

For the optimization model, there are mainly two decisive variables, namely the initial weight of a rule and the beliefs of scales in the conclusion part. The optimization objective is the error between the actual diagnostic category and the BRB diagnostic category, which would be calculated by the interval-based distance error [14].

(9) $$\begin{array}{cc} \min & E(\theta_{l},\beta_{n,l}) \end{array}$$

s. t.

(9a) $$0<\theta_{l}\leq 1$$

(9b) $$0\leq \beta_{n,l}\leq 1$$

(9c) $$\sum_{n=1}^{N}\beta_{n,l}=1$$

where Equation (9a) denotes that the initial weight of any rule should be (0, 1]. Equation (9b) denotes that the belief of any scale in any rule should be [0, 1]. Equation (9c) denotes that the sum of the beliefs of all scales in any rule should be equal to 1, since there is no incomplete information in this study. Note that more restraints can be added to the optimized model by including new parameters as decisive variables.

**Figure 1:**
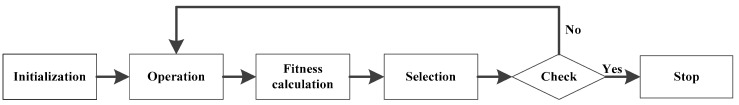
Optimization algorithm with evolutionary algorithms (EAs) as the optimization engine.

Figure 1 shows the optimization algorithm with evolutionary algorithms (EAs) as the optimization engine. For the optimization algorithm, there are multiple steps, including **initialization,** which defines the parameters for the optimization engine, e.g., the number of individual, the number of generations, etc., and the parameters of BRB, e.g., the number of rules/attributes/scales, the weights of initial rules and attributes, etc.; **optimization operation,** which depends on the specific EA as the optimization engine, e.g., crossover and mutation by the genetic algorithm (GA), the inertia weight and the contraction coefficient by the particle swarm optimization (PSO), etc.; **fitness calculation,** which should be referred to the inferencing procedure of BRB as in Section 3.2, i.e., the fitness value is normally the error between the actual thyroid nodule diagnosis result and that produced by BRB, e.g., if the actual diagnosis results is 4A and BRB also produces 4A, then the error is “0”; **selection,** which selects the optimal individuals to enter the next generation, i.e., an individual with the smallest (largest) fitness value if the fitness function is a minimization (maximization) function; and **stop criterion check** by the preset number of generations.

## 4. Belief Rule-Based Thyroid Nodule Diagnosis Approach under Three Assumptions

Based on the introduction and literature review, three assumptions and corresponding handling techniques are applied in this study.

### 4.1. Assumption 1: Deletion

**Assumption** **1:** *The clinical reports of thyroid nodule diagnosis with missing features are inadmissible by diagnostic approaches.*

Under Assumption 1, the clinical reports with missing features are incomplete, which should be deleted since they are inadmissible by the diagnostic approaches. There are mainly two deletion methods, namely listwise deletion and pairwise deletion [22]. When a case is missing a feature on at least one of the criteria, the listwise deletion method deletes it from the specific analysis [21]. Comparatively, the pairwise deletion method states that if the features of the paired criteria are missing, the cases containing these paired criteria are deleted when performing the statistical calculation of the paired criteria.

In short, the deletion method is to delete the clinical reports with missing features. The deletion method has the advantages of simplicity and being easy to operate under practical conditions. It is suitable when there are a large number of clinical reports and the missing features are relatively small.

### 4.2. Assumption 2: Imputation Based on the Distribution

**Assumption** **2:** *The missing features in clinical reports follow the same distribution of the respective criterion.*

Under Assumption 2, the missing features concerning one criterion could be replaced by a distribution of features with respective probabilities. The distribution of features would be derived by calculating the statistical distribution of the features concerning the specific criterion using all of the clinical reports. This is called imputation based on distribution. The steps of imputation based on distribution with three steps are as follows:

Step 1: Calculate the number of missing features for each criterion.

For *I* clinical reports with *M* criteria, calculate the number of clinical reports with a missing feature on *m*th criterion, $I_{m}^{miss}$ for *m* = 1, 2, …, *M*, and those containing a feature of the *m*th criterion, $I_{m}^{contain}$.

Step 2: Calculate the probability of each feature concerning each criterion.

For the *m*th criterion, the distribution of probabilities, *p*($A_{m}^{k}$), *k* = 1, 2, …, *K*, *m* = 1, 2, …, *M*, is calculated by using all clinical reports that contain a respective feature, $I_{m}^{contain}$, as Equation (10):

(10) $$p(A_{m}^{k})=num(A_{m}^{k})/I_{m}^{contain}$$

where $A_{m}^{k}$ denotes the *k*th feature on the *m*th criterion; *k* = 1, 2, …, *K*, *m* = 1, 2, …, *M*, *num*($A_{m}^{k}$) denotes the number of $A_{m}^{k}$ in $I_{m}^{contain}$.

Step 3: Replace missing features by the distribution of features with respective probabilities.

For the *m*th criterion, replace the missing features with the corresponding distribution of features with respective probabilities, as Equation (11):

(11) $$x_{m}=[(A_{m}^{1},p(A_{m}^{1})),(A_{m}^{2},p(A_{m}^{2})),\ldots ,(A_{m}^{K},p(A_{m}^{K}))]$$

where $x_{m}$ denotes the imputed features by the *m*th criterion, and there is $\sum_{k=1}^{K}p(x_{m}^{k})=1$.

Table the fourth criterion “calcification” with five features, namely $A_{4}^{1}$, $A_{4}^{2}$, $A_{4}^{3}$, $A_{4}^{4}$, and $A_{4}^{5}$, as an example. A total of 720 clinical reports have been collected, of which 331 clinical reports are without features on the “calcification” criterion and 389 are with features on the “calcification” criterion. Upon calculation based on 389 clinical reports, the numbers of features in the “calcification” criterion are num($A_{4}^{1}$) = 13, num($A_{4}^{2}$) = 3, num($A_{4}^{3}$) = 26, num($A_{4}^{4}$) = 4, num($A_{4}^{5}$) = 343, respectively. The probabilities of the features using the “calcification” criterion would be *p*($A_{4}^{1}$) = 0.0334, *p*($A_{4}^{2}$) = 0.0077, *p*($A_{4}^{3}$) = 0.0668, *p*($A_{4}^{4}$) = 0.0102, *p*($A_{4}^{5}$) = 0.8817, respectively. As a result, the missing features on the “calcification” criterion in the 331 clinical reports would be imputed as *x*_4_ = [($A_{4}^{1}$, 0.0334), ($A_{4}^{2}$, 0.0077), ($A_{4}^{3}$, 0.0668), ($A_{4}^{4}$, 0.0102), ($A_{4}^{5}$, 0.8817)].

### 4.3. Assumption 3: Benign by Default

**Assumption** **3:** *The doctors left the benign features unrecorded on purpose since they consider benign features irrelevant to the final diagnosis.*

The clinical reports of the patient after the ultrasound examination include multiple criteria, including margin, contour, echogenicity, calcification, vascularity, etc., each of which contains several different features [13]. Each criterion may contain several different features. In the diagnostic process, the diagnostic time for the doctors is very limited. Thus, the doctor should provide the overall diagnosis of the patient in a short time [14]. In this process, the malignant features are more decisive to provide the overall diagnosis than the benign features. As a consequence, radiologists may pay more attention to observing and recording the malignant features rather than some benign features, which will result in missing features in the clinical reports. Under this assumption, the missing features should be considered benign features instead of unknown ones.

In terms of benign by default, radiologists can concentrate more on analyzing the most decisive malignant features, which could help achieve the final diagnosis with higher accuracy. For example, if a patient is presenting several malignant symptoms and the rest are benign, the doctor may only record the most prominent malignant features and recommend further tests directly, which would result in certain missing features. By doing so, doctors can concentrate more on analyzing the most decisive malignant features, which could help achieve the final diagnosis with higher accuracy. Now consider another extreme condition. If a patient presents one highly malignant feature concerning one criterion and no other malignant features concerning other criteria at all, the doctor is assertive towards a malignant diagnosis. Then, such a malignant diagnosis would be made in the clinical report along with the malignant feature while leaving other benign features unrecorded. Under this condition, the clinical report would still be accurate and effective, but with multiple missing features.

### 4.4. The Belief Rule-Based Thyroid Nodule Diagnosis Approach

**Figure 2:**
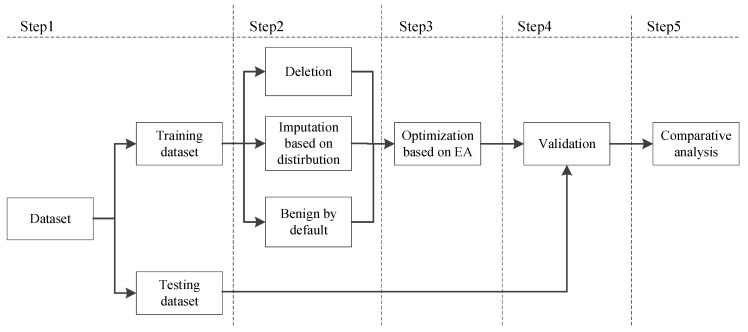
Thyroid nodule diagnosis approach using BRB under different assumptions.

According to three different situations, which are deletion, imputation based on distribution, and benign by default, an approach to diagnose thyroid nodules is proposed using BRB. The approach shown in Figure 2 includes five steps as follows.

Step 1: Gather data and divide them into training and testing datasets, respectively.

Step 2: Identify the corresponding assumption. Identify one out of the three assumptions, namely deletion, imputation, and benign. Details of the assumptions can be found in Section 4.1, Section 4.2, and Section 4.3, respectively.

Step 3: Construct the initial diagnostic model and further conduct parameter learning. Certain parameters are identified, the initial model is constructed, and then the model is further optimized under different assumptions using the training dataset. The detailed procedures for constructing an optimization model and designing a corresponding optimization algorithm can be found in Section 3.3, and the distance-based modeling error calculation procedure can be found in [14].

Step 4: Perform validation using the testing dataset.

Step 5: Gather validation results and perform further analysis, including focused analysis on each doctor and comprehensive analysis using the results of the ten doctors.

## 5. Thyroid Nodule Diagnosis Case Study

### 5.1. Background

In thyroid nodule diagnosis, five criteria are considered, namely margin, contour, echogenicity, calcification type, and vascular distribution. Doctors need to consider the features of the five criteria presented in the images to make an overall diagnosis (OD). Based on TIRADS classifications, which are widely accepted and used in many hospitals all around the world [7], thyroid nodules are often classified into eight categories, namely TIRADS 1, 2, 3, 4A, 4B, 4C, 5, and 6. Among the eight degrees, TIRADS 1 and 2 are considered highly benign, and TIRADS 6 is confirmed malignant. Thus, in this study, only TIRADS 3, 4A, 4B, 4C, and 5 are considered and listed in Table 1. The features concerning the five criteria in the clinical reports are recorded in the form of linguistic terms. Then, to be recognized by the proposed diagnostic model, they are translated into the corresponding TIRADS categories using the mapping relationship introduced in [14].

Table 2 and Table 3 show examples of features before and after translation. Based on Table 2 and Table 3, it can be found that there are a lot of missing features in the clinical reports. This may be caused by varied reasons, and the following case study would further explore how to deal with such missing features under three assumptions, namely deletion, imputation, and benign.

### 5.2. Statistical Analysis of Collected Cases of Thyroid Nodules

**Figure 3:**
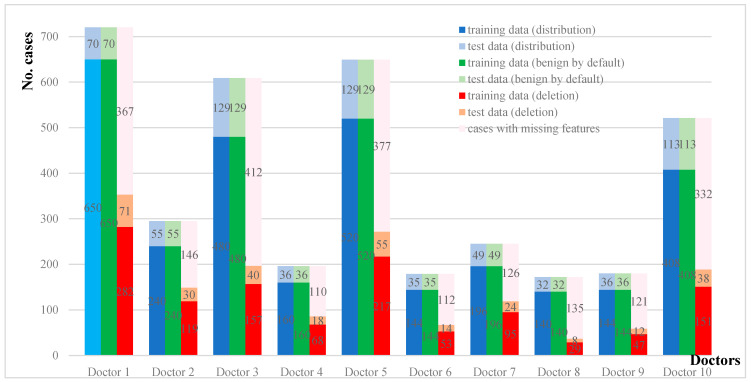
Training and test data under three assumptions of each doctor.

As shown in Figure 3, this study collected a total of 3766 clinical reports (also referred to as cases in the following of this case study) of 10 doctors on the diagnosis of thyroid nodules from January 2012 to February 2019. Among them, 3082 cases (from January 2012 to December 2017) were used as training data and 684 cases (from January 2018 to February 2019) were used as testing data.

**Figure 4:**
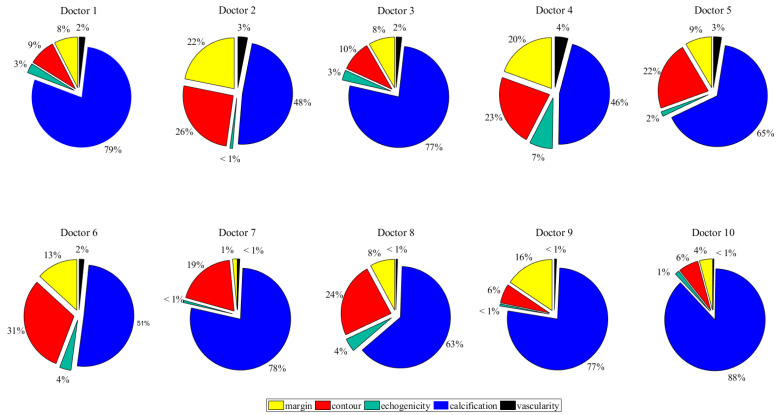
The percentage of criteria missing for each doctor.

In Figure 3, the first and second columns of each doctor’s histogram show the number of cases in the training/test datasets under the distribution and benign assumptions, respectively. Note that the training/testing datasets under the distribution and benign assumptions are entirely the same. The last column shows the number of cases in the training/testing datasets under the deletion assumption, as well as those with missing features that would not be used under the deletion assumption. We would further explore the makeup of the missing features for all of the ten doctors. Figure 4 presents the number of cases with missing features of the ten doctors.

In all cases with missing features, all of the ten doctors have the same feature mostly missing: calcification. For ten doctors, the cases with missing features on calcification make up about 36–69% of the entire cases and 46–88% of the cases with missing features. This is also consistent with the findings of many other doctors. Chan et al. reported that the absence of calcifications is nearly as common as their presence, which means that there exist some thyroid nodules without calcification features. It has also been reported that when faced with a possible benign calcification feature, doctors may not give a corresponding “calcification” diagnosis at all, for the reason that the presence of a feature of calcification may mistakenly increase the possibility of doctors considering the thyroid nodule as malignant. Considering that calcification is the criterion with the most missing features, it has great and pressuring challenges on how to meet this condition. To properly handle the missing features, we must explore different assumptions and handling techniques.

### 5.3. Results Comparison of One Doctor as an Example

In this section, Doctor No. 3 is used as an example to demonstrate the detailed procedures under the three assumptions, namely deletion, imputation, and benign. First of all, we would present the distance-based error between two TIRADS categories given in Table 4 using the error calculation procedure in Section 4.4.

Note that the distance-based error between the two TIRADS categories is different, ranging within [0, 1], which is more consistent with the actual situation. For example, the error (0.1943) between TIRADS 5 and TIRADS 4C is much smaller than that (0.9601) with TIRADS 3. Specifically, we would like to point out that the error (0.4957) between TIRADS 4B and TIRADS 5 is smaller than that (0.5148) with TIRADS 3. This is consistent with the practices in daily thyroid nodule diagnosis: TIRADS 4B is not a neutral diagnosis category but is in fact closer to a malignant diagnosis: TIRADS 4B would need to be confirmed by FNAB, as indicated by Table 1.

**Figure 5:**
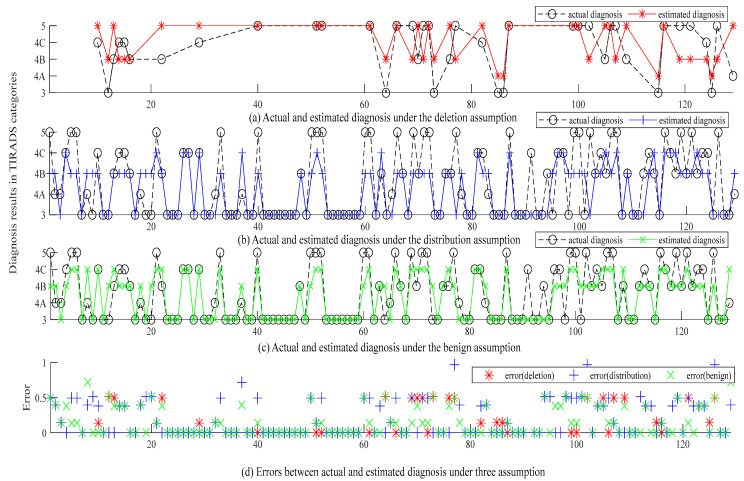
Actual output, estimated output, and error of Doctor No. 3 under three assumptions.

In this study, the differential evolutionary (DE) algorithm is applied as the optimization engine [39]. The individual is set at 20 and the number of generations is set at 500. A total of 30 runs were conducted for the three assumptions. Table 5, Table 6 and Table 7 show the three optimal BRBs produced using the data of Doctor No. 3 under the three assumptions. Figure 5 shows the diagnosis and error of the three BRBs whose MSEs are 0.2866 (deletion), 0.2014 (distribution), and 0.1539 (benign), respectively.

Based on Table 5, Table 6 and Table 7, and Figure 5, the following observations can be drawn:

(1) To compare the deletion and distribution assumptions, there are a total of 197 and 609 cases for constructing and optimizing the BRB under the deletion and distribution assumptions, respectively. Overall, the BRB under the deletion is inferior to that under the distribution assumption. However, upon further investigation, it is found that this may be due to the smaller testing dataset (only 40) under the deletion assumption than that (129) under the distribution assumption. If only comparing the 40 sets of data with complete features, the MSE for the BRB under the distribution assumption is 0.3023, which is actually inferior to that under the deletion assumption (0.2866). It indicates that the cases with missing features also contain very useful information and can help improve diagnostic accuracy.

(2) To compare the distribution and benign assumptions, the MSE (0.1539) of the benign assumption is smaller than that (0.2014) under the distribution assumption. For a total of 129 cases in the testing dataset, both models produced the same results for 87 cases. For the remaining 42 cases, the distribution assumption produced inferior results in comparison with the benign assumption: the distribution assumption produced bigger MSEs in 29 cases, while the benign assumption produced bigger MSEs in 13 cases.

(3) Moreover, the presented results also show varied tendencies among the three assumptions. The deletion assumption tends to produce more severe results, while the distribution and benign assumption tend to produce relatively less severe results.

### 5.4. Comparative Results of Ten Doctors under Three Assumptions

Following the proposed approach, three models under three assumptions are constructed and optimized for the remaining nine doctors. Table 8 shows the results of ten doctors under the three assumptions, as well as their average results concerning different assumptions and different doctors.

Based on Table 8, the following conclusions can be drawn:

(1) Generally, by comparing the average results of different assumptions, it is found that the benign assumption produced the smallest MSE of 0.1631, which is 11.32% smaller than that under the distribution assumption (average MSE is 0.1840) and 21.18% smaller than that under the deletion assumption (average MSE is 0.2118).

(2) Specifically, the above general observation can be different concerning different doctors. Comparing the results under the distribution and benign assumption, it can be found that for doctors 1/3/4/5/6/7/8, the results under the benign assumption are superior to those under the distribution assumption. Comparing the results under the deletion and benign assumptions, it can be found that for doctors 1/2/3/4/5/7/8, the results under the benign assumption are superior to those under the deletion assumption.

**Figure 6:**
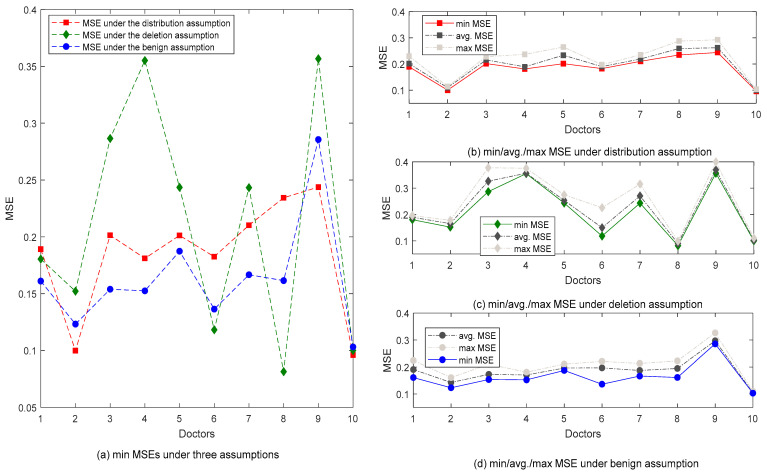
MSE comparison of ten doctors under three assumptions.

To summarize, both the general and specific comparative results show that the benign assumption prevails over the distribution assumption and further deletion assumption. However, it can also be found that the condition could vary concerning different doctors. Next, we would further explore the detailed results concerning the ten doctors. Figure 6a–d compares the MSEs under the three assumptions, which leads to the following conclusions.

(1) Overall, the benign assumption produced the optimal results with the smallest MSEs: five out of ten are with the smallest MSEs. The most inferior performance was achieved by the distribution assumption, and the deletion assumption produced the least optimal results. Nine out of 10 models under the benign assumption produced MSEs smaller than 0.2, while that number is 4 and 5 for the deletion and distribution assumptions, respectively.

(2) The distribution assumption produced the most stable results. According to the width of the band chart under the three assumptions presented in Figure 6b or c or d, the stability of the distribution assumption is the best, because the width of the band chart is relatively narrower for all of the ten doctors: the fluctuation between the maximum and the minimum values is smaller than the other two assumptions. The smallest fluctuation denotes that the data distribution of the training dataset is the same as that in the testing dataset, which is the natural result due to the adoption of the distribution assumption.

(3) Different doctors presented varied diagnostic tendencies towards different assumptions. Doctor 10 is the best in terms of both the stability and accuracy of the three assumptions. For Doctor 3, Doctor 6, and Doctor 7, their stabilities vary under different assumptions. For example, Doctor 6 has the poorest stability of the deletion assumption but also has a good stability of the distribution assumption. Moreover, it is worth mentioning that the results produced by Doctor 9 are relatively stable, but the accuracy is the most inferior under the three assumptions. As a consequence, specific recommendations can be made for different doctors as well. For example, it is recommended to apply the deletion assumption to Doctor 8 since their MSE under the deletion assumption is far smaller than the other two assumptions. Moreover, their models are also the most stable under the deletion assumption. Another example is Doctor 5, who should be recommended to adopt the benign assumption since their MSE under the benign assumption is the smallest and the corresponding models are also the most stable. In conclusion, different doctors have produced different tendencies. With this, different suggestions and guidance can be made to different doctors.

### 5.5. Discussion

Based on the results of the case study, the following can be observed:

(1) Missing features generally used in clinical reports in thyroid nodule diagnosis. Based on the statistical analysis of a total of 3766 clinical reports (cases) of ten doctors derived over 7 years, it is revealed that there are a large number of cases with missing features that could make up to between 50% and 80% of all cases. Among the five criteria critical to thyroid nodule diagnosis, calcification has the most missing features, ranging from 36% to 69% of all cases and from 46% to 88% of the cases with missing features. To properly handle the missing features, three assumptions are explored, namely deletion, which is the most prevailing practice; distribution, which replaces the missing features with a distribution of features; and benign by default, which replaces the missing features with benign features. Case study results show that:

(2) Upon comparison with the results under the three assumptions, it is found that the benign assumption produced the optimal results with the smallest MSE. That is to say, the missing features should be deemed benign features. It validates that the features are actually left unrecorded on purpose by many doctors because they consider benign features irrelevant to produce the final diagnosis. This is consistent with the daily practices of doctors but a little unorthodox with other practices in other domains. To further explain, missing features would normally be just considered missing and disregarded. If there is a large amount of data, the deletion approach would be fine. However, this is impractical in thyroid nodule diagnosis for at least one reason: there are not so much data in the first place: only 3766 sets of data have been collected from ten doctors over 7 years. The distribution assumption did not prove to be very inaccurate, but was quite impractical at least. This suggests that more information does not necessarily guarantee more accurate results; the information doctors record may be enough to obtain the accurate diagnostic result.

(3) Different doctors present varied tendencies. Although it validates that the benign by default assumption produced the optimal result, the actual condition varies depending on specific doctors. For example, Doctors 9 and 10 have the best performance under the distribution assumption, and Doctors 6 and 8 have the best accuracy under the deletion assumption. The difference concerning the tendencies demonstrated by the ten doctors is consistent with common knowledge and perceptions as well: people are just different. This novel difference can provide insights: the benign assumption can be applied if there are insufficient data, and the most appropriate assumption can be suggested to certain doctors if more information concerning this specific doctor is available.

(4) Guidelines for practical words can be drawn based on the results of this study. First, there is actually no need to compulsorily require the doctor to record all features because it is most accurate to just assume the missing features as benign by default. Second, the request for more comprehensive information may not lead to more accurate results but can at least improve model stability, since the distribution assumption produced models with the highest stability denoted by the smallest variance. Third, every doctor has respective tendencies towards different assumptions. Their diagnostic tendencies should be taken into consideration when providing specific suggestions for improving their diagnostic accuracy.

## 6. Conclusions

The uncertainty caused by missing features is a norm in data-driven medical decision making. By adopting the BRB to construct a model, three assumptions and corresponding handling techniques were compared, namely deletion, imputation based on distribution, and benign by default. A real-world thyroid nodule diagnosis case was studied for testing the three assumptions using a total of 3766 cases of ten doctors derived over 7 years from a tertiary hospital in the Anhui province. Case study results show that: (1) the conventional deletion assumption does not work as expected, mainly because deleting too many cases would cause losing too much useful information; (2) the imputations based on distribution assumption produced the most stable results, although slightly inferior overall performance, mainly suggesting that it may be impractical under daily medical practices; (3) the benign assumption produced the optimal results with the smallest MSEs for most doctors, which indicates that doctors do omit benign features which they consider irrelevant to the final diagnostic result; and (4) besides the overall results, different doctors also presented varied tendencies which can be used as guidance for their respective future work. Based on the above results, guidance for practical work has been suggested.

For future work, the benign assumption should be further tested on more diseases and enlarged benchmarks. Moreover, other white-box approaches or their combination with deep learning approaches could also be tested as the inference engine as well for identifying the fittest model for other medical cases.

## Figures and Tables

**Table 1:** TIRADS categories in this study.

Categories (Cat.)	Findings	Cancer Risk	Recommendations	Cancer on Surgery
TIRADS 3	probably benign	<3	Follow-up/FNAB (fine-needle aspiration biopsy)	1.79%
TIRADS 4A	low suspicion	3–24	Follow-up/FNAB	5.88%
TIRADS 4B	intermediate suspicion	25–75	FNAB	62.82%
TIRADS 4C	high suspicion	76–95	FNAB	91.22%
TIRADS 5	suggestive of malignancy	>95	FNAB	98.85%

**Table 2:** Examples of features in the clinical reports.

No	Margin	Contour	Echogenicity	Calcification	Vascular
1			slightly heterogeneous echo	circular strong echo	circular blood flow signals
2	relatively well-defined margin	relatively regular margin	mixed echo		punctate and bar blood flow signals
3	well-defined margin	regular margin	hypoecho	punctate strong echo	punctate and bar blood flow signals
4	ill-defined margin	slightly irregular margin	heterogeneous hypoecho	strong echoic plaques	abundant blood flow signals
5	well-defined margin	regular margin	heterogeneous hypoecho		circular blood flow signals

**Table 3:** Examples of translated clinical reports in TIRADS category.

No	Margin	Contour	Echogenicity	Calcification	Vascular
1			4B	4B	4A
2	4A	4A	3		4A
3	3	3	4B	5	4A
4	4B	4B	4C	5	3
5	3	3	4C		4A

**Table 4:** Distance-based error between two TIRADS categories.

		3	4A	4B	4C
		[0, 0.02]	[0.03, 0.25]	[0.26, 0.75]	[0.76, 0.95]
3	[0, 0.04]	0	0.1448	0.5148	0.8468
4A	[0.05, 0.25]	0.1448	0	0.0283	0.6733
4B	[0.26, 0.65]	0.5148	0.0283	0	0.4191
4C	[0.66, 0.95]	0.8468	0.6733	0.4191	0

**Table 5:** BRB of Doctor No. 3 under the deletion assumption.

No.	Weight	Criteria					OD Categories				
		Margin	Contour	Echo	Cal	Vascular	3	4A	4B	4C	5
1	0.8938	(3, 1)	(3, 1)	(3, 1)	(3, 1)	(3, 1)	0.5060	0.1043	0.2291	0.0693	0.0913
2	0.7849	((4B, 0.8300), (4C, 0.1700))	((4A, 0.0979), (4B, 0.9021))	((4C, 0.3145), (5, 0.6855))	((3, 1594), (4A, 8406))	((4A, 0.3465), (4B, 0.6535))	0.1358	0.1251	0.3642	0.1417	0.2332
3	0.9421	(5, 1)	(5, 1)	(5, 1)	(5, 1)	(5, 1)	0.0924	0.1608	0.2368	0.4075	0.1025

**Table 6:** BRB of Doctor No. 3 under the distribution assumption.

No.	Weight	Criteria					OD Categories				
		Margin	Contour	Echo	Cal	Vascular	3	4A	4B	4C	5
1	0.8674	(3, 1)	(3, 1)	(3, 1)	(3, 1)	(3, 1)	0.0014	0.4371	0.4229	0.1082	0.0304
2	0.3781	((4B, 0.7793), (4C, 0.2207))	((4A, 0.1379), (4B, 0.8621))	((4C, 0.0551), (5, 0.9449))	((4A, 0.6357), (4B, 0.3643))	((3, 0.8411), (4A, 0.1589))	0.2515	0.0000	0.0405	0.2760	0.4320
3	0.4261	(5, 1)	(5, 1)	(5, 1)	(5, 1)	(5, 1)	0.0493	0.1830	0.2316	0.0602	0.4760

**Table 7:** BRB of Doctor No. 3 under the deletion assumption.

No.	Weight	Criteria					OD Categories				
		Margin	Contour	Echo	Cal	Vascular	3	4A	4B	4C	5
1	0.9409	(3, 1)	(3, 1)	(3, 1)	(3, 1)	(3, 1)	0.3968	0.0332	0.2858	0.0422	0.2420
2	0.8351	((4A, 0.0719), (4B, 0.9281))	((4C, 0.7377), (5, 0.2623))	((4B, 0.6935), (4C, 0.3065))	((4A, 0.5079), (4B, 0.4921))	((3, 0.3750), (4A, 0.6250))	0.1639	0.0859	0.2413	0.3277	0.1812
3	0.7926	(5, 1)	(5, 1)	(5, 1)	(5, 1)	(5, 1)	0.0335	0.1546	0.3422	0.4235	0.0462

**Table 8:** MSEs for ten doctors under the three assumptions.

Doctor No.	Deletion	Distribution	Benign	Avg.
1	0.1805	0.1891	0.1611	0.1769
2	0.1521	0.0999	0.1232	0.1251
3	0.2866	0.2014	0.1539	0.2140
4	0.3552	0.1811	0.1524	0.2296
5	0.2435	0.2012	0.1874	0.2107
6	0.1182	0.1825	0.1364	0.1457
7	0.2433	0.2102	0.1666	0.2067
8	0.0815	0.2344	0.1615	0.1591
9	0.3567	0.2438	0.2856	0.2954
10	0.1000	0.0959	0.1031	0.0997
Avg.	0.2118	0.1840	0.1631	0.1863

## Data Availability

The data used to support the findings of this study are available from the corresponding author upon request.
